# Antifibrotic treatment response comparison of progressive pulmonary fibrosis and idiopathic pulmonary fibrosis

**DOI:** 10.55730/1300-0144.5866

**Published:** 2024-05-27

**Authors:** Berna Akıncı ÖZYÜREK, Kerem ENSARİOĞLU, Tuğçe Şahin ÖZDEMİREL, Esma Sevil AKKURT, Özlem ÖZDAĞ, Esma ZENBİLLİ

**Affiliations:** Department of Chest Disease, Atatürk Sanatorium Research and Training Hospital, University of Health Sciences, Ankara, Turkiye

**Keywords:** Idiopathic pulmonary fibrosis, progressive pulmonary fibrosis, antifibrotic treatment, progression, prognosis

## Abstract

**Background/aim:**

Idiopathic pulmonary fibrosis (IPF) and progressive pulmonary fibrosis (PPF) are two entities categorized as fibrotic lung diseases. With a similar clinical presentation and treatment modalities in many cases, the line differentiating these two diseases may not be evident. Hence, it was aimed herein to evaluate the effectiveness of antifibrotic treatment and the course of fibrotic lung diseases.

**Materials and methods:**

The study included patients diagnosed with IPF and PPF who were given antifibrotic treatment and followed-up for 12 months at our clinic. At the final follow-up, treatment response and radiological evaluation were investigated via high-resolution computed tomography.

**Results:**

Eighty-seven patients were included in the study (57 with IPF and 30 with PPF). Under antifibrotic treatment, there were no statistically significant decreases in the six-minute walking test, forced vital capacity, and diffusing capacity of the lungs for carbon monoxide values at 6 and 12 months posttreatment. The most common side effects were photosensitivity for patients under the pirfenidone regimen, while diarrhea was predominantly observed in the PPF group. Radiological progression was observed in 22.9% of the patients at 12 months posttreatment. Hospitalization requirements were more evident in the PPF group, with at least one hospitalization history present in 60% (n = 18) of the PPF patients compared to 12.3% (n = 7) of the IPF patients.

**Conclusion:**

A personalized approach is preferred with similar clinical profiles for both treatment modalities, with specific side effects considered.

## 1. Introduction

Idiopathic pulmonary fibrosis (IPF) is a chronic, progressive fibrosing interstitial pneumonia of unknown cause that is associated with radiological and histologic features of usual interstitial pneumonia (UIP) [1]. IPF is the most prevalent type of idiopathic interstitial pneumonia, which occurs most commonly in advanced age. Progressive pulmonary fibrosis (PPF) is defined as a clinical entity different than IPF in the ATS/ERS/JRS/ALAT Clinical Practice Guideline 2022. However, some clinical similarities are evident between IPF and PPF, as both are defined by deteriorating lung function, a suboptimal response to immunomodulatory treatments, and early mortality. In IPF and PPF, an inappropriate lung repair mechanism involves common downstream mechanisms that eventually lead to pulmonary fibrosis.

Pirfenidone and nintedanib are the two antifibrotic medications that are, under specific conditions, recommended for interstitial lung disease (ILD) treatment. They both have similar effect profiles in clinical settings. Nintedanib is currently the preferred treatment modality in PPF due to the available studies stating successful disease control. Studies on the role of pirfenidone in PPF are currently being conducted [2,3].

Studies comparing IPF and PPF treatment responses to antifibrotic regimens remain limited in the literature. This study aimed to evaluate the effectiveness of antifibrotic treatment and the course of the diseases in both groups.

## 2. Materials and methods

### 2.1. Patient selection

The study population consisted of patients receiving antifibrotic treatment between January 1st, 2021, and January 15th, 2022, diagnosed with either IPF or PPF, at the pulmonary medicine clinic of a tertiary hospital. Treatment history was confirmed from the hospital computer records, which included relevant drug reports. Demographic characteristics (age, sex, body mass index (BMI), concomitant diseases, medication history), results of pulmonary function test, and six-minute walking test (6MWT) values at baseline, and 6 and 12 months posttreatment were recorded. High-resolution computed tomography was requested at 12 months posttreatment to evaluate treatment response, and radiological evaluation was also included. Additionally, any comorbidities were noted.

### 2.2. Comorbidity evaluation

Any known cardiac comorbidity, including coronary arterial disease history, arrhythmia diagnosis, or heart failure of any etiology, was classified under cardiovascular disease history. Obstructive sleep apnea diagnosis was only accepted after confirmation by a sleep center study report. Malignancies of any etiology, including pulmonary-originated ones, were defined and grouped under malignancy history. The patients’ BMI was utilized for weight assessment, with a BMI over 25 being defined as overweight, a value lower than 18 being defined as underweight, and other values in between being considered normal. The occupational exposure definition consisted of patients who had stated a relevant exposure history that could be attributed to an active or former occupation. This definition excluded patients who had been diagnosed with occupation-related pulmonary disease.

### 2.3. Inclusion criteria

The study included patients diagnosed with either IPF or PPF between the mentioned dates, according to the guidelines available at the time of diagnosis [1]. The main inclusion criterion was a minimum duration of 12 months under antifibrotic treatment.

### 2.4. Exclusion criteria

Patients who refused medical treatment during the follow-up period and whose radiology, PFT, and prognosis information could not be accessed from the hospital system or patient files were excluded from the study.

### 2.5. Antifibrotic initiation criteria

Antifibrotic treatment was initiated for the patients according to the latest guidelines at the time of diagnosis [4]. The current guidelines did not deviate in terms of diagnosis from the former ones, with antifibrotic initiation being recommended for patients with typical UIP findings at IPF. The radiological presentation that is appropriate for typical UIP includes subpleural involvement, traction bronchiectasis, honeycombing, and the exclusion of findings that would indicate the presence of another interstitial disease. PPF was defined as the presence of at least two of three criteria (worsening symptoms, radiological progression, and physiological progression) in a patient with an ILD other than IPF within the previous year with no alternative explanation [4]. The patients grouped under the PPF classification were mostly those receiving additional treatment up to the time of evaluation and then were later evaluated for the need for antifibrotic treatment. Off-label approval was obtained for nintedanib and pirfenidone in the PPF group, and patients antifibrotic treatment was initiated.

### 2.6. Statistical evaluation

Statistical analysis was performed on IBM SPSS Statistics for Windows 25.0 (IBM Corp., Armonk, NY, USA) after initial data collection was done on Microsoft Excel (Microsoft Corp., Redmond, WA, USA). All parameters were first investigated with descriptive analysis, in which the mean ± standard deviation (SD) were used to express the parametric values, while median, and 25th and 75th percentile values were used to express the nonparametric values. To assess whether a result was distributed parametrically or not, histogram charts were primarily used along with Kolmogorov–Smirnov analysis for confirmation when required. IPF and PPF parameters were given under their respective groups and as a combined value for reference. Parameters deemed specific for one subgroup (such as PPF diagnostic criteria) were not evaluated further and were given as descriptive results. For values deemed parametric, comparisons between two groups were made using the independent samples T-test after evaluation with the Shapiro–Wilk’s test for linearity and confirmed by the Levene’s test for equality of variance. For nonparametric results, the Mann–Whitney U test was used for comparison.

## 3. Results

A total of 87 patients were included in the study, of whom 57 were diagnosed with IPF, and the remaining 30 were diagnosed with PPF. The average age of the patients was 66.6 (±7) years. The IPF group was predominantly male (male-to-female ratio = 54:3), while the PPF group had a more balanced distribution (male-to-female ratio = 14:16). Four patients in the PPF group and 43 in the IPF group received pirfenidone (Table 1).

The average 6MWT for the IPF group was 407.7 (±116.3) m, while it was 353.8 (±83.6) for the PPF group, with a reduced walking distance observed in both groups at six and 12 months posttreatment. The PPF group had an overall higher forced vital capacity (FVC) at the time of diagnosis, at 78% (±15.7) compared to 72.3% (±17.8) in the IPFs group. A similar result was noted for the diffusing capacity of the lungs for carbon monoxide (DLCO), with PPF and IPF group values of 64.3% (±11.7) and 58.9% (±18.2) DLCO, respectively. The side effects of the drugs at follow-up are shown in detail in Table 2. Hospitalization requirement was greater in the PPF group, with at least one hospitalization history present in 60% (n = 18) of patients compared to 12.3% (n = 7) of the IPF patients. Drug cessation due to side effects was less required in the PPF group (n = 9, 30%) and observed at a rate of 24.6% (n = 14) in the IPF group. Radiological progression was observed in 22.9% (n = 20) of all the patients at 12 months posttreatment (Table 2). Drug cessation among the IPF patients was divided according to etiology for the subgroup evaluation. Photosensitivity and disease progression (with additional drug switch) were the main reasons for drug cessation, observed in 12.3% (n = 7) and 8.8% (n = 5) of patients, respectively. The majority of the IPF patients were diagnosed radiologically (n = 43, 75.4%). In the subtype analysis of PPF, most patients were classified under non-UIP pattern radiologically (n = 25, 83.3%), and fibrotic hypersensitivity pneumonia (F-HP) and fibrotic nonspecific interstitial pneumonia (F-NSIP) were observed as the two dominant types. A total of 6 patients had been diagnosed with an autoimmune disease related to PPF. All the PPF patients had a treatment regimen with glucocorticoid before antifibrotic initiation. The patients had an average duration of 33.1 months before the diagnosis of PPF and initiation of antifibrotic treatment, with 43.3% (n = 13) being under concomitant glucocorticoid treatment in addition to antifibrotic treatment (Table 3). There was no difference between age, smoking history and duration, BMI, FVC, and total side effects between the IPF and PPF groups (Table 4).

## 4. Discussion

The IPF and PPF patients had statistically relevant differences, as seen in the study. The 6MWT result was lower in the PPF group compared to the IPF group, although both groups had a lower overall result at 12 months posttreatment. An interesting observation was an increase in the 6MWT values at 6 months posttreatment and in the FVC and DLCO results at 12 months posttreatment in the IPF group. This unexpectedly impressive treatment response, while valid in itself, was a result that is expected to disappear over a longer follow-up duration. It strengthens the rather high morbidity of PPF patients, as despite being under similar treatment, no such response was observed among them. This observation was further supported by the loss of pulmonary function in the PPF group despite having higher initial test results than the IPF group. A relatively higher hospitalization requirement for the PPF group was also evident. These changes may be attributed to the progressive nature of PPF being different than IPF, and also to a myriad of different comorbidities causing PPF, thus leading to a different clinical outcome despite having a similar clinical presentation. Drug adherence was also another factor of note, as the IPF group had a higher rate of drug switching compared to the PPF group, whose treatment modalities had at least one additional immunosuppressive agent already present before antifibrotic treatment, mainly glucocorticoids. These observations lead to the assumption that, while IPF patients may initially have worse performance evaluation compared to PPF patients, the progressive nature of PPF may eventually cause a worse clinical status, despite patients being under multiple treatment modalities.

The risk of IPF is increased by current and past smoking history. Smoking also contributes to an overall worse clinical presentation. In the current study, a smoking history was common, with 74.7% (n = 65) and an average of 23.8 packs/year, consistent with the literature [5]. There was no difference in age, smoking history, and BMI between the IPF and PPF groups. However, there was an evident difference regarding gastroesophageal reflux (GER) between the IPF and PPF patients, which could be attributed to an overall increased incidence of GER among the IPF population and GER may have been asymptomatic in the PPF group.

The study in the USA identified some occupations associated with IPF. These professions include agriculture, animal husbandry, hairdressing, bird breeding, stone cutting, and polishing, which cause exposure to organic and inorganic materials. Lung microbiome disruption due to factors like viral infections has been shown to have a negative prognostic factor effect in studies [6]. In the present research, occupational rate of exposure history was around 20% in both groups.

The exact prevalence of PPF remains unknown. In a real-world cohort of patients with ILD, 25% of fibrosing ILD (excluding IPF) had a progressive phenotype [7]. Another study found that 18% and 32% of non-IPF ILDs had a progressive fibrosing phenotype [8]. PPF, in itself, is not a diagnosis but rather a description of a clinical progress with subtypes including idiopathic nonspecific interstitial pneumonia, fibrotic hypersensitivity pneumonitis, connective tissue disease-associated ILD, unclassifiable fibrotic ILD, sarcoidosis, and ILD related to occupational exposures [9–11]. This further complicates the differences between PPF and IPF.

Studies have shown that comorbidities are significantly higher in IPF compared to the general population, with reported respiratory comorbidities being chronic obstructive pulmonary disease (COPD), lung cancer, pulmonary embolism, and pulmonary hypertension. In the current study, the majority of the patients had a known comorbidity (n = 59, 67.8%), with cardiovascular disease and hypertension being the most common comorbidities in both groups.

Pirfenidone and nintedanib are antifibrotic drugs used in the treatment of IPF. Antifibrotic drugs have been shown to help slow disease progression, reduce lung function loss, and improve survival. Pirfenidone is a medication with antifibrotic and antiinflammatory properties, although the exact mechanism of action is still unclear. In a metaanalysis of nine randomized controlled trials (1824 IPF patients) evaluating the use of pirfenidone in the treatment of IPF, pirfenidone reduced the risk of IPF progression or death by 35%, and significantly improved lung function, including delaying vital capacity and FVC decline, compared with a control group [12]. Nintedanib is a tyrosine kinase inhibitor that affects growth factor receptors, including vascular endothelial growth factor, fibroblast growth factor, and platelet-derived growth factor [13]. An evaluation of the INPULSIS and INSTAGE studies stated that nintedanib had the same effect on FVC reduction in patients, with a similar safety and tolerance compared to the pirfenidone group [14].

Progressive fibrosing ILDs have been traditionally treated with corticosteroids and immunosuppressive therapies. It is possible that immunosuppressive and corticosteroid treatments are insufficient and ineffective, indicating the need for an effective cure [15]. Antifibrotic treatment is utilized to prevent the natural course of the disease, which otherwise would have progressed to severe fibrosis [16]. Nintedanib and pirfenidone have been suggested as possible treatment choices due to similarities in the pathobiological pathways causing fibrosis between IPF and PPF [17]. The SENSCIS trial demonstrated the effectiveness of nintedanib among patients with ILD related to systemic sclerosis. INBUILD trial was performed to evaluate efficacy and safety of nintedanib among patients with PF-ILD other than IPF. This trial showed that nintedanib decreased the rate of FVC decline and disease progression regardless of the underlying ILD subtype, which led to further research into its impact on PPF progression. In the most recent IPF-PPF guidelines, nintedanib was given a conditional recommendation for treating PPF [18].

The RELIEF research was performed to evaluate the effectiveness of pirfenidone in PPF; however, it was terminated early due to insufficient recruitment [19]. The analysis performed with the available data favored pirfenidone arm over the placebo [19]. Current studies recommend further studies regarding pirfenidone and its role on non-IPF ILD [1]. In the current study, nintedanib was the preferred drug for most of the PPF patients (86.7%, n = 26), as the drug was given FDA approval and reimbursed for PPF treatment within the country’s healthcare system.

The main side effects of pirfenidone are gastrointestinal intolerance and photosensitivity. The same side effect profile regarding gastrointestinal intolerance applies to nintedanib, with diarrhea being the most observed. In the current study, regarding side effect evaluation, an average of 72.4% of patients had at least one reported drug-related side effect. The most often reported side effect in the IPF group was photosensitivity, while the most frequent side effects for the PPF group were diarrhea and nausea. Drug cessation due to side effects was less required in the PPF group 30%) and observed at a rate of 24.6% in IPF patients. Photosensitivity and disease progression (with additional drug switch) were the main reasons for drug cessation, observed at 12.3% and 8.8% of patients, respectively. These findings were also found to be parallel with the literature data.

Compared to IPF, the PPF patients had more yearly hospitalizations, which was attributed to F-HP being the most commonly observed PPF etiology. It was assumed that most of these patients either could not exclude the cause of hypersensitivity or the etiology of hypersensitivity was unknown, further increasing exposure and causing a more severe clinical outcome.

There is no head-to-head comparison study between these two drugs, and since both have similar effects on loss of function, there is no superiority in use [20]. The current study is the only one in Türkiye that compares IPF and PPF treatment outcomes.

A limitation of the study was that, as per the nature of PPF and its requirement to be treated, and the fact that IPF patients also require antifibrotic treatment so long it is tolerated, a control group with the exclusion of either drug regimen could not be followed for the study.

In conclusion, antifibrotic treatment appears to have a similar treatment profile and disease control on PPF despite being initially utilized for IPF. There is no significant difference in the side effect profile of the drugs in either disease; thus, the observed side effects reflect the drug profile more than the underlying disease itself. Considering these findings, initiating antifibrotic treatment immediately for IPF diagnosis and at an appropriate time for PPF confirmation is a valid approach for disease control. Antifibrotic treatment in the PPF is also justified regardless of the underlying diseases responsible for ILD, further strengthening the role of antifibrotics once the diagnosis is confirmed.

## Figures and Tables

**Table 1:** Demographic information, comorbidities and initial assessment.

		IPF (n = 57)	PPF (n = 30)	Total (n = 87)
**Age**	**Mean (±SD)**	66.6 (7)	68.5 (7)	67.3 (7)
**Sex**	**Male (n, %)**	54 (94.7)	14 (46.7)	68 (78.2)
	**Female (n, %)**	3 (5.3)	16 (53.3)	19 (21.8)
**Smoking history**	**Nonsmoker (n, %)**	13 (22.8)	9 (30)	22 (25.3)
	**Smoker (n, %)**	44 (77.2)	21 (70)	65 (74.7)
**Smoking package year**	**Mean (±SD)**	26.4 (19.46)	18.93 (16.21)	23.83 (18.6)
**Comorbidity evaluation (n, %)**				
**Comorbidity presence**	**Absent**	21 (36.8)	7 (23.3)	28 (32.2)
	**Present**	36 (63.2)	23 (76.7)	59 (67.8)
**Cardiovascular disease history**	**Absent**	44 (77.2)	21 (70.0)	65 (74.7)
	**Present**	13 (22.8)	9 (30.0)	22 (25.3)
**Hypertension**	**Absent**	51 (89.5)	18 (60)	69 (79.3)
	**Present**	6 (10.5)	12 (40)	18 (20.7)
**Diabetes mellitus**	**Absent**	52 (91.2)	13 (43.3)	65 (74.7)
	**Present**	5 (8.8)	17 (56.7)	22 (25.3)
**Gastroesophageal reflux**	**Absent**	41 (71.9)	30 (100)	71 (81.6)
	**Present**	16 (28.1)	0 (0)	16 (18.4)
**Obstructive sleep apnea**	**Absent**	50 (87.7)	30 (100)	80 (92)
	**Present**	7 (12.3)	0 (0)	7 (8)
**Chronic obstructive pulmonary disease**	**Absent**	49 (86)	30 (100)	79 (90.8)
	**Present**	8 (14)	0 (0)	8 (9.2)
**Malignancy history**	**Absent**	55 (96.5)	30 (100)	85 (97.7)
	**Present**	2 (3.5)	0 (0)	2 (2.3)
**Cerebrovascular disease**	**Absent**	56 (98.2)	30 (100)	86 (98.9)
	**Present**	1 (1.8)	0 (0)	1 (1.1)
**Initial assessment (n, %)**				
**BMI**	**Underweight**	17 (29.8)	6 (20)	23 (26.4)
	**Average**	34 (59.6)	23 (76.7)	57 (65.5)
	**Overweight**	6 (10.5)	1 (3.3)	7 (8)
**Occupational exposure**	**Absent**	38 (66.7)	21 (70)	59 (67.8)
	**Present**	11 (19.3)	8 (26.7)	19 (21.8)
	**Unknown**	8 (14.0)	1 (3.3)	9 (10.4)
**Desaturation at diagnosis**	**Absent**	54 (94.7)	23 (76.7)	77 (88.6)
	**Present**	3 (5.3)	7 (23.3)	10 (11.4)
**Antifibrotic choice**	**Pirfenidone**	43 (75.4)	4 (13.3)	47 (54)
	**Nintedanib**	14 (24.6)	26 (86.7)	40 (46)

**SD:** Standard deviation, **IPF:** Idiopathic pulmonary fibrosis, **PPF:** Progressive pulmonary fibrosis. Smoker definition includes both ex and current smokers.

**Table 2:** Respiratory function test results, side effects during follow-up and outcome.

Testing method (mean, ±SD)		IPF (n = 57)	PPF (n = 30)	Total (n = 87)
**6MWT distance (meter)**	**Diagnosis**	407.7 (116.3)	353.8 (83.6)	389.2 (108.8)
	**6. Month**	412.8 (96.5)	350.5 (76.3)	391.1 (94.4)
	**12. Month**	401.9 (126.3)	332 (78.1)	377.8 (116.5)
**Forced vital capacity (%)**	**Diagnosis**	72.3 (17.8)	78 (15.7)	74.4 (17.3)
	**6. Month**	71.5 (16.3)	75.8 (12.6)	73 (15.1)
	**12. Month**	74.2 (17.1)	74.3 (14)	74.2 (16)
**Diffusion capacity of lung for carbon monoxide (%)**	**Diagnosis**	58.9 (18.2)	64.3 (11.7)	60.9 (16.3)
	**6. Month**	56.9 (19.5)	65.6 (12.2)	60.1 (17.6)
	**12. Month**	57.4 (19.8)	62.7 (12.1)	59.3 (17.6)
**Side effect evaluation (n, %)**				
**Side effect presence**	**Absent**	17 (29.8)	6 (20)	23 (26.4)
	**Present**	40 (70.2)	23 (76.7)	63 (72.4)
**Photosensitivity**	**Absent**	48 (84.2)	30 (100)	78 (89.7)
	**Present**	9 (15.8)	0 (0)	9 (10.3)
**Diarrhea**	**Absent**	52 (91.2)	16 (53.3)	68 (78.2)
	**Present**	5 (8.8)	14 (46.7)	19 (21.8)
**Liver enzyme elevation**	**Absent**	49 (86)	24 (80)	73 (83.9)
	**Present**	8 (14)	6 (20)	14 (16.1)
**Dyspepsia**	**Absent**	46 (80.7)	30 (100)	76 (87.4)
	**Present**	11 (19.3)	0 (0)	11 (12.6)
**Nausea/vomiting**	**Absent**	56 (98.2)	20 (66.7)	76 (87.4)
	**Present**	1 (1.8)	10 (33.3)	11 (12.6)
**Acute renal failure**	**Absent**	54 (94.7)	30 (100)	84 (96.6)
	**Present**	3 (5.3)	0 (0)	3 (3.4)
**Fatigue**	**Absent**	51 (89.5)	30 (100)	81 (93.1)
	**Present**	6 (10.5)	0 (0)	6 (6.91)
**Dizziness**	**Absent**	52 (91.2)	30 (100)	82 (94.3)
	**Present**	5 (8.8)	0 (0)	5 (5.7)
**Weight loss**	**Absent**	57 (100)	28 (93.3)	85 (97.7)
	**Present**	0 (0)	2 (6.7)	2 (2.3)
**Rash**	**Absent**	57 (100)	28 (93.3)	85 (97.7)
	**Present**	0 (0)	2 (6.7)	2 (2.3)
**Appetite loss**	**Absent**	57 (100)	26 (86.7)	83 (95.4)
	**Present**	0 (0)	4 (13.3)	4 (4.6)
**Hospitalization within one year**	**Absent**	50 (87.7)	10 (33.3)	60 (69)
	**Present**	7 (12.3)	18 (60.0)	25 (28.7)
**Drug cessation due to side effects**	**Absent**	43 (75.4)	21 (70)	64 (73.7)
	**Present**	14 (24.6)	9 (30)	23 (26.3)
**Progression at first year**	**Absent**	42 (73.7)	25 (83.3)	67 (77.1)
	**Present**	15 (26.3)	5 (16.7)	20 (22.9)

**SD:** Standard deviation, **IPF:** Idiopathic pulmonary fibrosis, **PPF:** Progressive pulmonary fibrosis. Drug cessation refers to halting the initially given antifibrotic for a limited time and then continuing them after the symptoms recede.

**Table 3:** IPF drug cessation causes, PPF subtypes and treatment modalities.

IPF drug cessation etiology *(n, %)*		Total (n = 57)
**Photosensitivity**	**Absent**	50 (87.7)
	**Present**	7 (12.3)
**Progression**	**Absent**	52 (91.2)
	**Present**	5 (8.8)
**Liver Enzyme Elevation**	**Absent**	53 (93)
	**Present**	4 (7)
**Acute Renal Failure**	**Absent**	56 (98.2)
	**Present**	1 (1.8)
**Pancytopenia**	**Absent**	56 (98.2)
	**Present**	1 (1.8)
**PPF types and diagnosis parameters (n, %)**		**Total (n = 30)**
**Radiological pattern**	**UIP**	5 (16.7)
	**Non-UIP**	25 (83.3)
**Subtype**	**F-NSIP**	11 (36.7)
	**F-HP**	12 (40)
	**Autoimmune disease**	6 (20)
	**Sarcoidosis**	1 (3.3)
**Autoimmune disease subtype**	**Rheumatoid arthritis**	3 (10)
	**Systemic sclerosis**	2 (6.7)
	**Sjogren’s disease**	1 (3.3)
	**IPAF**	1 (3.3)
**Immunosuppressive treatment history**	**Glucocorticoid**	30 (100)
	**Azathioprine**	1 (3.3)
	**Mycophenolate mofetil**	2 (6.7)
	**Biological agent**	2 (6.7)
**PPF diagnostic criteria**	**FVC fall above 10%**	19 (63.3)
	**FVC fall between 5%–10%, and clinical or radiological progression**	16 (53.3)
	**Clinical and radiological progression**	25 (83.3)
**PPF concomitant treatment (n, %)**		**Total (n = 30)**
**Glucocorticoid**	**Absent (n, %)**	17 (56.7)
	**Present (n, %)**	13 (43.3)
**MycophenolateMofetil**	**Absent (n, %)**	27 (90)
	**Present (n, %)**	3 (10)
**Biological agent**	**Absent (n, %)**	29 (98.9)
	**Present (n, %)**	1 (1.1)
**PPF treatment duration (mean, 25–75** **^th^** **)**		
**Treatment duration before antifibrotic initiation (months)**		33.1 (12–40)
**Treatment duration after antifibrotic initiation (months)**		15.8 (12–18)

**IPF:** Idiopathic pulmonary fibrosis, **PPF:** Progressive pulmonary fibrosis, **UIP:** Usual interstitial pneumonia, **F-NSIP:** Fibrotic nonspecific interstitial pneumonia, **F-HP:** Fibrotic hypersensitivity pneumonia, **IPAF:** Interstitial pneumonia with autoimmune features, **FVC:** Forced vital capacity

**Table 4:** Independent samples T-Test for comparison between IPF and PPF patients.

	t	dF	p	%95 Confidence interval	
				Lower	Upper
**Age**	–1.153	85	0.252	−4.994	1.327
**Smoking history**	0.727	85	0.469	−0.125	0.269
**Smoking package year**	1.798	85	0.076	−0.132	0.276
**BMI**	−0.207	85	0.837	−0.279	0.227
**Occupational exposure**	−0.421	77	0.675	−0.242	0.157
**6MWT distance**					
**Initial** **6. month** **12. month**	2.248	85	**0.027**	6.226	101.620
	3.054	84	**0.003**	21.717	102.793
	2.761	85	**0.007**	19.580	120.279
**Forced vital capacity**					
**Initial** **6. month** **12. month**	−1.573	85	0.120	−6.100	3.879
	−1.243	82	0.217	−11.132	2.570
	−0.020	85	0.984	−7.307	7.163
**Total side effects**	−0.899	84	0.371	−0.294	0.111
**Drug cessation due to side effects**	−0.541	85	0.590	−0.254	0.145
